# Presidential Symposium: From Global to Local: An Age-Inclusive and Rights-Based Approach to Gerontological Education

**DOI:** 10.1093/geroni/igab046.1313

**Published:** 2021-12-17

**Authors:** Dana Bradley, Judith Howe

## Abstract

In June 2020, the Gerontological Society of America (GSA) joined in solidarity in the movement to condemn the entrenched racism undermining American society and build upon a gero-rich international historical base of supporting human rights. However, as gerontological educators, we need to expand on the conversation of racism to the broader global discussion of inclusivity and elimination of discrimination. A global focus on human rights of older persons, which began in 1982 at the World Assembly on Aging and has led to the current discussion of the proposed UN Convention on the Rights of Older Persons. The Academy of Gerontology in Higher Education (AGHE) is GSA's education group of colleges and universities that offers education, training, curricular innovations, and research programs in the field of aging. The work of this group is grounded in an age-inclusive and rights-based perspective, and members are committed to an international view demonstrated through AGHE’s tagline Global Leaders in Advancing Education on Aging; This symposium explores the role of age-inclusivity and a rights-based perspective in gerontology and geriatrics education and offers both challenges and best practices for moving forward. The first presentation explores the meaning of age-inclusivity in aging education in a global context and asks how do we build upon our international roots? Our second presenter shares a proposed framework for a rights-based approach to gerontology education. The third presentation explores an example of a rights-based training program. We conclude with a lively discussion focusing on how to take action through education.

